# Changes in periprosthetic bone mineral density after medial unicompartmental knee arthroplasty: a prospective cohort study

**DOI:** 10.1007/s00264-025-06711-0

**Published:** 2025-12-05

**Authors:** Liangsheng Wei, Qiaoning Yue, Chuanlin Zhang, Shaogang Miao, Xiang Jiang, Pei Liu, Xiguang Zhang, Yi Zhang

**Affiliations:** 1 Kunming Medical University, Kunming, China; 2 Department of Orthopedic, Sixth Affiliated Hospital of Kunming Medical University: People’s Hospital of Yuxi City, Yuxi, China; 3 Department of Orthopedic, Luoyang Orthopedic-Traumatological Hospital: Henan Luoyang Orthopedic Hospital, Luoyang, China

**Keywords:** Unicompartmental knee arthroplasty, Bone mineral density, Anteromedial osteoarthritis, Implants, Bone loss

## Abstract

**Background:**

Unicompartmental Knee Arthroplasty (UKA) is effective for knee anteromedial osteoarthritis (AMOA), but aseptic prosthetic loosening causes failures. While periprosthetic bone loss links to loosening in Total Knee Arthroplasty (TKA), this association and post-UKA periprosthetic Bone Mineral Density (BMD) changes are understudied. Systematically exploring dynamic post-UKA BMD changes is vital for optimizing management and reducing loosening risk.​.

**Patients and methods:**

This prospective study included 40 patients (40 knees) with knee AMOA who underwent UKA (January 2020–January 2024). All received cemented Oxford unicompartmental prostheses implanted by the same surgeon (standard technique). Dual-Energy X-ray Absorptiometry (DEXA) measured periprosthetic BMD preoperatively, and at one, three, six and 12 months postoperatively to analyze change patterns.​.

**Results:**

Periprosthetic BMD decreased rapidly at one and three months postoperatively, then increased at six and 12 months (*p* < 0.05). No significant differences were noted in tibial prosthesis BMD changes (ROI 1, ROI 2) or femoral prosthesis stem posterior BMD values (ROI 4) between six and 12 months (*p* > 0.05).​.

**Conclusion:**

Early postoperative (≤ 3 months) rapid periprosthetic BMD decline in UKA suggests potential clinical value of early anti-osteoporotic treatment.

**Level of evidence:**

Level 2b - Prospective case-control study.

## Introduction

Unicompartmental Knee Arthroplasty (UKA), characterized by minimal trauma, preservation of knee proprioception, and maintenance of motor function, has been validated through years of clinical practice as an effective treatment for anteromedial knee osteoarthritis (AMOA). In recent years, the global annual volume of UKA procedures has increased by 15%–20%, establishing it as an important treatment option for knee osteoarthritis (KOA) [1, 2]. However, UKA still has a mid-term surgical failure rate of 5%–10%. Data from a 13-year follow-up of the New Zealand Joint Registry indicate that aseptic prosthetic loosening is the second leading cause of UKA revision (accounting for 23%), second only to the progression of osteoarthritis in the contralateral compartment [3]. A study by Karin B van Dorp et al. [4] on the Oxford Phase III unicompartmental prosthesis also found that the failure rate caused by aseptic loosening reached 8.7% at five years postoperatively, implying that abnormal periprosthetic bone mass may be a key factor affecting the long-term efficacy of UKA [5].

In Total Knee Arthroplasty (TKA), the association between periprosthetic bone loss and aseptic loosening has been widely confirmed: Kyoun Kim et al. [6] found that periprosthetic BMD decreased by 18% at three months after TKA, and patients with bone loss exceeding 20% had a three fold higher risk of long-term loosening; Lewis PL et al. [7] confirmed via histopathological studies that stress shielding-induced osteocyte apoptosis is the core mechanism of periprosthetic bone loss after TKA. Nevertheless, UKA and TKA differ substantially in prosthesis design (unicompartmental vs. total compartmental) and stress distribution (local weight-bearing vs. total joint weight-bearing) [8]. This raises critical questions: Does the pattern of post-UKA periprosthetic BMD change mirror that of post-TKA? And does the association between periprosthetic BMD changes and aseptic loosening apply to UKA? Currently, relevant research remains limited: Mahmut Tuncer et al. [9] included only 11 UKA patients, with a small sample size and no control group; Richmond BI et al. [10] reported in a two year follow-up that proximal tibial BMD was well-preserved after UKA but did not analyze femoral-side changes or influencing factors.

This study measured periprosthetic BMD preoperatively, and at one month, three months, six months, and one year post-UKA, aiming to clarify the dynamic pattern of BMD changes after UKA and provide evidence-based support for optimizing postoperative management strategies for UKA.

## Materials and methods

### Patient selection

This study enrolled patients who underwent primary unilateral unicompartmental knee arthroplasty (UKA) for anteromedial knee osteoarthritis (diagnosed via clinical and imaging examinations) at our hospital between January 2020 and January 2024, with a final cohort of 40 patients completing full follow-up (Table 1). Inclusion criteria were scheduled UKA and willingness to participate in postoperative follow-up, while exclusion criteria included severe bone metabolic diseases or use of drugs affecting bone mineral metabolism within six months preoperatively; female patients had no menopausal status restrictions. All UKAs were performed by the same senior orthopaedic surgeon (with > 15 years of joint surgery experience) via a medial parapatellar approach, using Oxford bone-cement unicompartmental prostheses and adhering to the standard Oxford unicompartmental technique—including tibial and femoral osteotomies and cemented prosthesis fixation. Postoperatively, all patients were permitted immediate full weight-bearing and followed a unified rehabilitation protocol; no contralateral non-prosthetic knees were used as controls, and only longitudinal comparisons of periprosthetic bone mineral density (BMD) in the same patient at different time points were conducted. This study was approved by the Ethics Committee and Institutional Review Board of our hospital (Approval No. 2025kmykdx6f273), all enrolled patients provided written informed consent after fully understanding the study purpose, methods, and potential risks, and the study was conducted in strict compliance with the Declaration of Helsinki.

**Table 1:** Baseline characteristics of the patients

Characteristic	Value (SD)
Number of patients	40
Male/female	11/29
Age (years)	63 (7.1)
Body mass index (kg/m^2^)	26.5 (2.7)

### ROI division and BMD measurement

#### Equipment and calibration

A GE LUNAR Prodigy DEXA bone densitometer was used, with daily phantom calibration (the calibration phantom was the standard phantom supporting the equipment, with a standard BMD value of 1.056 g/cm² and a measurement error ≤ 0.5%) to ensure the accuracy of the equipment.

#### ROI division

**Fig. 1:**
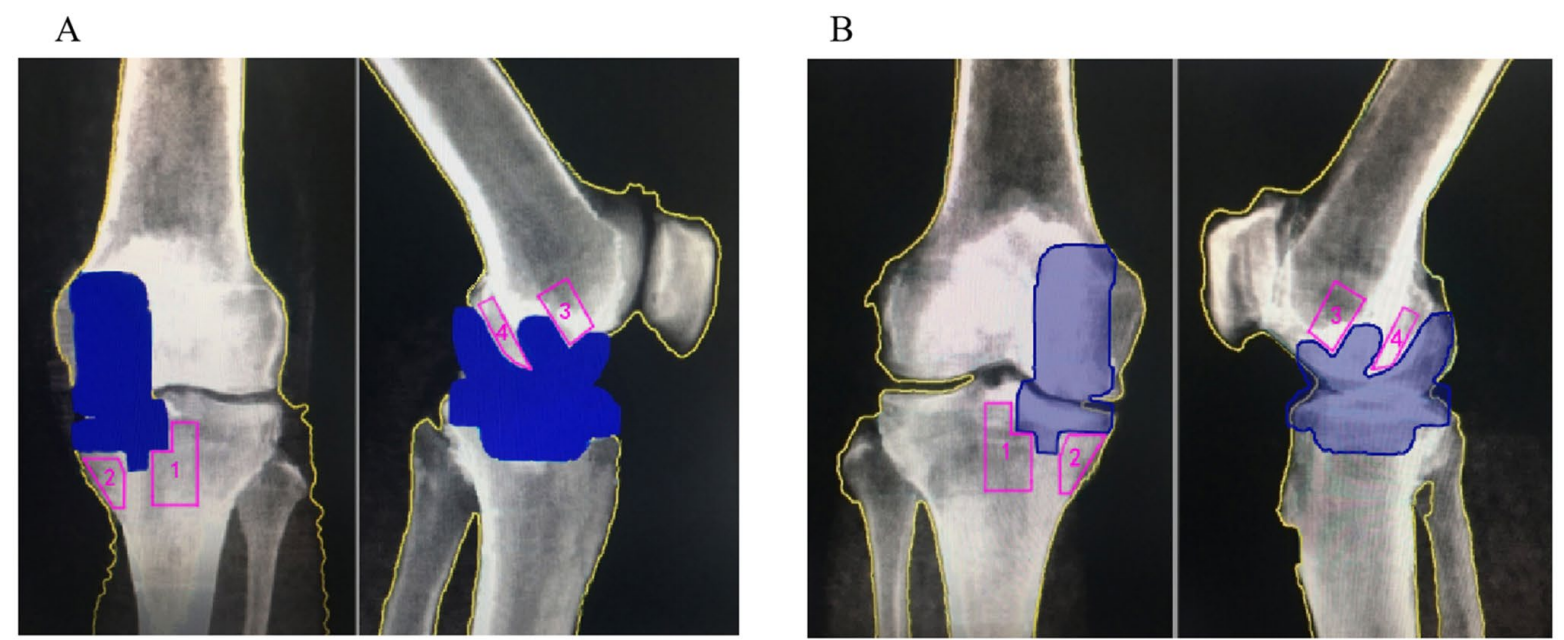
The location of ROI sinantero-posterior DEXA scans. **A**: Operated knee. **B**: Unoperated control knee. A diaphysial midline was used to divide the medial (ROI1) and lateral (ROI2) ROIs. ROI 1 was the lateral side of the tibial prosthesis keel, and ROI 2 was the medial side of the tibial prosthesis keel. The ROI around the femoral prosthesis was divided using lateral knee radiography and was also divided into two ROIs: ROI 3 before the femoral prosthesis column and ROI 4 after the femoral prosthesis column

Anteroposterior knee radiographs were used for dividing ROIs around the tibial prosthesis, which were divided into 2 ROIs: ROI 1 was the lateral side of the tibial prosthesis keel, and ROI 2 was the medial side of the tibial prosthesis keel (Fig. 1A); lateral knee radiographs were used for dividing ROIs around the femoral prosthesis, which were also divided into 2 ROIs: ROI 3 was the anterior aspect of the femoral prosthesis stem, and ROI 4 was the posterior aspect of the femoral prosthesis stem (Fig. 1A).

All ROIs were divided in accordance with the same standard. For preoperative ROI division, postoperative images of the same patient at the same scale were referenced to ensure that the ROIs were the same region at all time points before and after surgery (Fig. 1B), while avoiding bone cement, prostheses, osteophytes, and sclerotic bone .

#### BMD measurement

A DEXA bone densitometer (GE LUNAR) was used to measure the BMD of periprosthetic regions of interest (ROIs) of the knee joint preoperatively, and at one month, three months, six months, and one year postoperatively.

Preoperative radiographs were taken in standard anteroposterior and lateral positions: for the anteroposterior position, the patient was in the supine position with both knees in the standard anteroposterior position and feet together, fixed with a dedicated phantom; for the lateral position, the patient was in the lateral decubitus position with the knee slightly flexed, the lateral edge of the knee close to the examination table, and the patella perpendicular to the examination table.

Postoperative measurement positions were adjusted slightly based on the standard anteroposterior and lateral positions: for the anteroposterior position, adjustments were made to align the keel of the tibial prosthesis in a straight line; for the lateral position, adjustments were made to ensure the lateral side of the prosthesis faced the X-ray projection direction directly. The radiography range included the femoral condyles, patella, and tibial tuberosity, covering approximately 15 cm above and below the knee joint as the center.

Each ROI was divided in accordance with the above-established protocol, and Orthopaedics software was used to calculate the BMD of each ROI (unit: g/cm²). Measurements were performed by 3 physicians in accordance with the same standard, and the average value was taken as the result. The results measured by this method were defined as “actual values”. The “change value” was calculated by subtracting the actual value at the previous time point from the actual value at the subsequent time point (e.g., the change value at 1 month postoperatively was the actual value at one month postoperatively minus the preoperative actual value, and the change value at 1 year postoperatively was the actual value at 1 year postoperatively minus the actual value at 6 months postoperatively).

### Statistical analysis

SPSS 25.0 software was used for data analysis. Normality and homogeneity of variance tests were conducted. According to data characteristics, statistical analyses were performed using repeated-measures analysis of variance, Friedman test, and paired samples t-test/Wilcoxon signed-rank test. A *p*-value < 0.05 was considered statistically significant.

## Results

A total of 40 patients (40 knees) with anteromedial osteoarthritis (AMOA) who underwent cemented Oxford unicompartmental knee arthroplasty (UKA) completed the one year follow-up, with no loss to follow-up; baseline demographic and clinical characteristics of the cohort are summarized in Table 1, all patients received standard postoperative care, and no major perioperative complications were reported during the follow-up period.

### Inter-ROI differences in periprosthetic BMD at different postoperative time Points​

Periprosthetic BMD values (g/cm²) of ROIs 1–4 and inter-ROI statistical comparisons are summarized in Table 2 (chronological order below). Preoperatively, BMD values were 1.005 ± 0.159 (ROI 1), 1.057 ± 0.160 (ROI 2), 1.220 ± 0.212 (ROI 3), 1.164 ± 0.238 (ROI 4); repeated-measures ANOVA showed significant inter-ROI differences (*p* < 0.05), with post-hoc analysis (e.g., Tukey’s HSD) confirming ROI 3 > ROI 4 > ROI 2 > ROI 1. At 1 month postoperatively, BMD slightly decreased (ROI 1: 0.950 ± 0.151; ROI 2: 1.003 ± 0.124; ROI 3: 1.165 ± 0.202; ROI 4: 1.108 ± 0.208); consistent with baseline, repeated-measures ANOVA still showed significant inter-ROI differences (*p* < 0.05), and post-hoc tests verified the same ranking. By 3 months, BMD further declined (ROI 1: 0.798 ± 0.098; ROI 2: 0.811 ± 0.143; ROI 3: 0.926 ± 0.175; ROI 4: 0.902 ± 0.174); repeated-measures ANOVA still detected overall inter-ROI differences (*p* < 0.05), but post-hoc tests showed no difference between ROI 3 and ROI 4 (*p* > 0.05). By 6 months, BMD recovered (ROI 1: 0.922 ± 0.151; ROI 2: 0.976 ± 0.123; ROI 3: 0.975 ± 0.168; ROI 4: 0.936 ± 0.169), and inter-ROI differences completely disappeared (*p* > 0.05). At 1 year, BMD exceeded baseline (ROI 1: 1.052 ± 0.146; ROI 2: 1.116 ± 0.163; ROI 3: 1.212 ± 0.185; ROI 4: 1.188 ± 0.178); repeated-measures ANOVA confirmed reappeared inter-ROI differences (*p* < 0.05), but post-hoc tests showed no differences between ROI 1 vs. ROI 2 or ROI 3 vs. ROI 4 (both *p* > 0.05).​.

**Table 2:** Periprosthetic BMD Values (g/cm2 Mean ± SD) of ROIs 1–4 at Different Time Points

Time Point	ROI 1	ROI 2	ROI 3	ROI 4	Repeated-Measures ANOVA (F, *p*-value)	Post-hoc Inter-ROI Hierarchy (Bonferroni Correction)
Preoperative	1.005 ± 0.159	1.057 ± 0.160	1.22 ± 0.212	1.164 ± 0.238	F = 12.86, *p* < 0.001	ROI 3 > ROI 4 > ROl 2 > ROI 1 (all *p* < 0.05)
1 month post-op	0.95 ± 0.151	1.003 ± 0.124	1.165 ± 0.202	1.108 ± 0.208	F = 10.32, *p* < 0.001	ROI 3 > ROI 4 > ROI 2 > ROI 1 (all *p* < 0.05)
3 months post-op	0.798 ± 0.098	0.811 ± 0.143	0.926 ± 0.175	0.902 ± 0.174	F = 8.75, *p* < 0.001	(ROI 3 ≈ ROI 4) >(ROI 2 > ROI 1) (*p* < 0.05)
6 months post-op	0.922 ± 0.151	0.976 ± 0.123	0.975 ± 0.168	0.936 ± 0.169	F = 1.89, *p* = 0.135	No significant inter-ROI differences
1 year post-op	1.052 ± 0.146	1.116 ± 0.163	1.212 ± 0.185	1.188 ± 0.178	F − 9.56, *p* < 0.001	(ROI 3 ≈ ROI 4)>(ROI 2 ≈ ROI 1) (*p* < 0.05)

(Note: “≈”indicates no significant difference; post-hoc analysis for significant *p*-values)

### Temporal dynamics of BMD in each individual ROI​

**Fig. 2:**
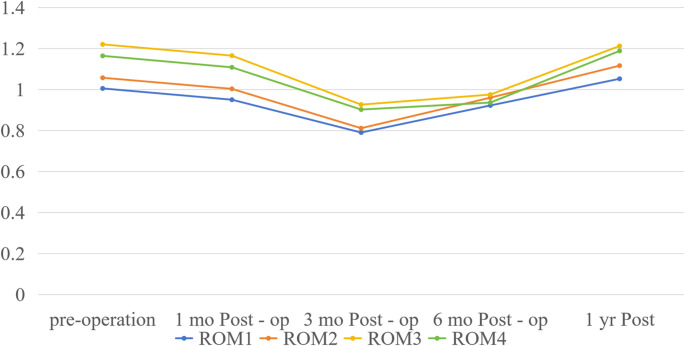
BMD values (g/cm^2^) of ROIs 1–4 at various time points before and after surgery

Temporal BMD trends for ROIs 1–4 (with Friedman test and post-hoc results) are detailed in Table 3; Fig. 2. All ROIs shared a three-phase pattern: early postoperative decrease → trough at three months → recovery (exceeding baseline by 1 year), with specific dynamics as follows.

**Table 3:** Temporal BMD Changes (g/cm^2^ Mean ± SD) and Statistical Results for ROIs 1—4

ROI	Preop	1M Post-op	3M Post-op	6M Post-op	1Y Post-op	Friedman *p*-value	Pairwise Significant Differences (*p* < 0.05)	BMD Change Magnitude (g/cm^2^, vs. previous time point)
1	1.005 ± 0.159	0.950 ± 0.151	0.798 ± 0.098	0.922 ± 0.151	1.052 ± 0.146	< 0.05	All time points	-0.055→-0.152→+0.124→+0.130
2	1.057 ± 0.16	1.003 ± 0.124	0.811 ± 0.143	0.976 ± 0.123	1.116 ± 0.163	< 0.05	All time points	-0.063→-0.171→0.148→+0.149
3	1.22 ± 0.213	1.165 ± 0.202	0.926 ± 0.175	0.975 ± 0.168	1.212 ± 0.185	< 0.05	All time points	-0.056→ -0.238→+0.049→+0.237
4	1.164 ± 0.238	1.108 ± 0.208	0.902 ± 0.174	0.936 ± 0.169	1.188 ± 0.178	< 0.05	All except 6M vs. 1Y	-0.056→-0.206→+0.034→+0.252

(Note: “1M/3M/6M/1Y Post-op”=1/3/6/12 months postoperatively; change magnitude: negative = decrease, positive = increase)

For ROI 1,preoperative BMD 1.005 ± 0.159, decreased by 0.055 ± 0.059 at 1 month, further fell by 0.152 ± 0.101 at 3 months (trough), then recovered (0.124 ± 0.094 at 6 months, + 0.130 ± 0.085 at 1 year, surpassing baseline). Friedman test showed significant differences across all time points (*p* < 0.05), and post-hoc comparisons (e.g., Wilcoxon-Bonferroni) confirmed all pairs were significant (all *p* < 0.05).

ROI 2 followed an identical trend: preoperative BMD 1.057 ± 0.160, decreased by 0.063 ± 0.085 (1 month) and 0.171 ± 0.099 (3 months), then recovered (0.148 ± 0.095 at 6 months, + 0.149 ± 0.132 at 1 year); consistent with ROI 1, Friedman test and post-hoc results were significant (all *p* < 0.05).

For ROI 3, preoperative BMD 1.220 ± 0.213, decreased by 0.056 ± 0.052 (1 month) and 0.238 ± 0.101 (3 months), then recovered modestly (0.049 ± 0.047 at 6 months) and substantially (+ 0.237 ± 0.122 at 1 year); Friedman test and post-hoc results were significant (all *p* < 0.05).

For ROI 4, preoperative BMD 1.164 ± 0.238, decreased by 0.056 ± 0.058 (1 month) and 0.206 ± 0.098 (3 months), then recovered (0.034 ± 0.052 at 6 months, + 0.252 ± 0.127 at 1 year); Friedman test showed overall significance (*p* < 0.05), but post-hoc tests showed no difference between 6 months and 1 year (*p* > 0.05), with other pairs significant (all *p* < 0.05).

## Discussion

This study dynamically monitored periprosthetic BMD before and after UKA. Compared with existing research, its ROI division focused on periprosthetic bone regions critical for prosthetic stability, avoided interference from prostheses, bone cement, sclerotic bone, and osteophytes, and ensured consistent pre- and post-op ROI selection. A key finding was that BMD in all periprosthetic ROIs decreased at one to three months post-op; while the exact timing of the BMD trough is unclear, a significant upward trend emerged around six months, continuing until one year, with final BMD exceeding preoperative levels.

Regarding the mechanism of post-knee arthroplasty periprosthetic bone loss, Kyoun Kim et al. [6], Lewis PL et al. [7], and van Loon CJ et al. [8] highlighted early postoperative mechanical factors, with stress shielding as the primary driver (inhibiting osteocyte proliferation, inducing apoptosis, and reducing BMD, with severity proportional to stress shielding), a view validated by van Lenthe GH et al. [11] via 3D finite element modeling. Beyond stress shielding, Antti JAROMA et al. [12] suggested contributions from periprosthetic bone metabolism, bone remodeling, and lower limb alignment changes, while Anett Mau-Moeller et al. [13] proposed involvement of other unknown factors.​.

This study aligns with the general pattern of post-knee arthroplasty periprosthetic bone loss reported previously, though differences in change magnitude and timing were noted—potentially due to variations in ROI division, sample size, or ethnicity. The recovery of BMD to above preoperative levels at one year may relate to improved knee OA symptoms and increased daily activity, which enhance mechanical stimulation of periprosthetic bone and promote bone formation.​.

A limitation of this study is the absence of aseptic prosthetic loosening in follow-up patients, precluding analysis of the relationship between BMD changes and loosening. However, the observed early postoperative BMD reduction suggests timely, effective early post-UKA anti-osteoporotic (OP) treatment may have positive therapeutic value. Future studies with longer follow-up and larger samples are needed to further explore the BMD-loosening association and validate the efficacy of early anti-osteoporotic intervention.​.

## Data Availability

No datasets were generated or analysed during the current study.
